# A168 COSTS OF MISSED WORK AMONG PEOPLE WITH INFLAMMATORY BOWEL DISEASE: A CROSS-SECTIONAL POPULATION-REPRESENTATIVE STUDY

**DOI:** 10.1093/jcag/gwac036.168

**Published:** 2023-03-07

**Authors:** E Kuenzig, M Lebenbaum, J Mason, M V Vyas, G G Kaplan, S K Murthy, L E Targownik, E I Benchimol

**Affiliations:** 1 Child Health Evaluative Sciences, SickKids Research Institute; 2 SickKids Inflammatory Bowel Disease Centre, Division of Gastroenterology, Hepatology and Nutrition, The Hospital for Sick Children; 3 Canadian Centre for Health Economics; 4 Institute of Health Policy, Management and Evaluation, University of Toronto; 5 The Centre for Addiction and Mental Health; 6 ICES; 7 St. Michael's Hospital - Unity Health Toronto, Toronto; 8 Medicine & Community Health Sciences, University of Calgary, Calgary; 9 Medicine, University of Ottawa; 10 Ottawa Hospital Research Institute; 11 The Ottawa Hospital IBD Centre; 12 School of Epidemiology and Public Health, University of Ottawa, Ottawa; 13 Mount Sinai Hospital Centre for Inflammatory Bowel Disease, Department of Medicine; 14 Paediatrics, University of Toronto, Toronto, Canada

## Abstract

**Background:**

The relapsing and remitting symptoms experienced by people with inflammatory bowel disease (IBD), including abdominal pain and diarrhea, may impact their employability and workplace productivity.

**Purpose:**

Report the impact of IBD on missed work and its associated costs using population-representative data with the goal of understanding the population-level indirect costs of IBD in Canada.

**Method:**

We used the 2010 and 2014 waves of the Canadian Community Health Survey (CCHS) to compare missed work and its associated costs among survey respondents aged 20 to 64 years with physician-diagnosed IBD compared to those without IBD. Students and those with non-IBD bowel disorders were excluded. The cost of missed work was derived from reported annual income and the number of workdays missed. Survey weights were used for descriptive statistics. Poisson regression modified for binary outcomes was used to compare employment within the past 3 months among those with and without IBD. Heckman models (two-stage models accounting for the likelihood of being employed) were used to compare the number of days missed and the cost of missed work in the two groups. Models were adjusted for age, sex, education, marital status, immigration status, visible minority status, number of non-IBD chronic conditions, and mood or anxiety disorder. All costs were inflation-adjusted to 2022 Canadian dollars.

**Result(s):**

Among 67,907 eligible respondents, 810 (weighted 1.0%) had IBD. Employment in the last 3 months was reported among 70% of people with IBD compared to 80% of people without IBD (RR 0.92, 95% CI 0.88 to 0.96). Among those employed in the prior 3 months, employed people with IBD missed an average of 1.6 (SD 4.4) days of work compared to 1.0 (SD 3.5) days missed among those without IBD, corresponding to $415 (SD 1315) in lost wages among those with IBD and $235 (SD 932) among those without IBD. Accounting for the higher rates of unemployment in those with IBD using Heckman models, people with IBD missed an additional 1.1 days (95% CI 0.7 to 1.5) of work and lost an additional $270 (95% CI 163 to 377) in income over 3 months. Extrapolating to the estimated 184,460 employed Canadians with IBD, the attributable indirect cost of missed work due to IBD is nearly $200 million annually.

**Conclusion(s):**

The economic burden of IBD resulting from missed work is substantial. Since IBD is often diagnosed during adolescence and early adulthood, employability and productivity are important long-term patient-centered outcomes. Novel interventions that could mitigate these attendant effects of IBD are urgently needed to improve patient outcomes.

**Please acknowledge all funding agencies by checking the applicable boxes below:**

None

**Disclosure of Interest:**

None Declared

